# Effect of forest management on tree diversity in temperate ecosystem forests in northern Mexico

**DOI:** 10.1371/journal.pone.0233292

**Published:** 2020-05-18

**Authors:** Jose Carlos Monarrez-Gonzalez, M. Socorro Gonzalez-Elizondo, Marco Antonio Marquez-Linares, Pedro Joaquin Gutierrez-Yurrita, Gustavo Perez-Verdin

**Affiliations:** 1 Instituto Nacional de Investigaciones Agropecuarias y Forestales, Campo experimental Valle del Guadiana, Durango, Mexico; 2 Instituto Politécnico Nacional, CIIDIR DGO, Durango, Mexico; 3 Instituto Politécnico Nacional, CIIEMAD, Ciudad de Mexico, Mexico; Oregon State University, UNITED STATES

## Abstract

An important challenge for silvicultural practices is the conservation of tree diversity while fulfilling the traditional objectives of forest management, most notably timber harvesting. The purpose of this study was to compare the tree diversity before and after the application of silvicultural treatments in a temperate forest in northern Mexico. Fifteen experimental plots, each measuring 2500 m^2^, were established to evaluate the immediate effect of four silvicultural treatments. These treatments were identified by their levels of management: intensive (clearcut, removal 100%), semi-intensive (removal of 59–61% of basal area), conservative (removal of 29–31% of basal area), and a control group. New forest guidelines, in contrast to conventional approaches, were applied to the semi-intensive and conservative treatments based on health and diversity conditions. Basal area, canopy cover, tree and total volume were measured in each plot. The Importance Value Index, alpha diversity, and evenness were estimated before and after treatments. Eighteen species belonging to five genera and five families were found in the study area. The species with the highest ecological values were *Pinus durangensis*, *P*. *teocote*, *Quercus sideroxyla*, and *Quercus convallata* with IVI numbers between 13.6 and 24.5%. Alpha diversity was intermediate (Margalef: 2.9 to 3.8), while dominance and evenness were above average compared to other studies (Simpson: 0.69 to 0.77; Shannon-Wiener: 1.44 to 1.6; Pielou: 0.76 to 0.85). The species evenness index in the conservative treatment was high (Sorensen, Jaccard, quantitative Sorensen and Morisita-Horn; 88 to 99%), although abundance decreased. Overall, there were no significant differences in IVI values and diversity indicators before and after treatments, with the exception of the clearcut treatment. When associating the diversity indices with stand variables, only the Pielou’s evenness index showed a significant relationship between them. We concluded that both the conservative and semi-intensive treatments did not generate significant changes in tree diversity, but the former had slightly higher alpha diversity indices. These results can provide a better insight on silvicultural practices and their effects on species composition.

## Introduction

An ecosystem is defined as the group of organisms and their physical environment that interact in a site [1]. Biodiversity is the variability of living organisms and the ecological complexes in which they exist [2]. An ecosystem and its biodiversity are closely related, as the latter is a structural characteristic of the former, and the variability among ecosystems is an element of biodiversity [3]. Forest ecosystems provide, in a simultaneous, dynamic, and complex way, a great variety of services [4]. Ecosystem services are the benefits that people receive from nature [3]. They include climate regulation, water quality, food, wood, and recreation, among others. Naturally, changes in biological diversity can affect the services that these ecosystems offer [4]. Plant diversity has multiple roles in the provision or regulation of various ecosystem services. A greater diversity implies a greater potential for producing various ecosystem services and functions at multiple times and places, as well as a greater resilience to perturbance [5–7]. For instance, there is a positive effect of plant diversity on provisioning of useful plant products, erosion control, resistance to plant invasions, and pathogen regulation [8]. It is still unclear the optimal number of species needed to maintain ecosystem services [7], which strongly depends on the ecosystem and region. Determining that number will require years of research in different ecosystems, management contexts, as well as at different temporal and spatial scales [7, 8]. One ecosystem of particular research interest is the temperate forests of northern Mexico that are managed through various silvicultural regimes.

Forest management involves the decisions and activities that take place for the proper use, conservation, and promotion of forest resources, while simultaneously trying to satisfy the present and future needs of society [9]. The composition and abundance of tree species are primary attributes of forest ecosystems that are affected by silvicultural practices [10, 11]. Forest management practices create stand structures that have an influence on species' diversity and richness. These intrinsically associated interactions are complex; therefore, different ecosystems and variety in management systems produce ecosystem functions and services that are just as diverse [7, 12–14].

The effect that silvicultural treatments have on tree diversity is determined by the intensity of the treatment and the succession stage of the forest [6, 15]. Thus, conservation of tree diversity is a condition that can be manipulated through forest management to keep these ecosystems productive [16]. Intensive silvicultural treatments, including commercial plantations, clearcuts, and retention (seed trees), produce low values of diversity indices. In contrast, individual selection of trees or light-intensity thinnings appear to favor diversity [17]. Several authors have found that tree diversity increases the productivity of stands [15–17]. Selection cuts (either individual or group-based cuts), in particular, have a positive effect on species diversity of the understory compared to intensive regeneration cuts (clearcuts, seed trees) [15]. Also, forest productivity increases significantly in forests that have an assortment of tree species compared to single-species stands [13]. Nevertheless, there are authors who have suggested that forest management has positive and negative effects on tree diversity, or no effect at all [18, 19]. These contrasting results can be explained by the effects of the ecological group, succession stages, type of management, and the timing and area of disturbance.

Forests with timber harvesting, that underwent changes in the composition of tree species, had the greatest effect on species richness [18, 20]. Maintaining the diversity of tree species and their positive effects on the ecosystem function, at the stand level, is a challenge faced today in temperate forests [21]. In the cases of forests managed by their owners, traditional management objectives are mainly oriented to increase economic profitability in the short and mid-term. Forest planning to produce only timber does not consider the interactions among other ecosystem services and may cause degradation of forest structures [9, 20, 22]. The importance of having forest systems that conserve tree diversity and fulfill the traditional objectives of forest management has prompted the need to evaluate the impact that current management practices have on the conservation of diversity and production of ecosystem services [23].

In Mexico, the temperate forest ecosystem represents about 17% (34 million ha) of the country (195 million ha) [24]. In terms of plant diversity, these forests host around 10,000 species, practically a third of the national flora [25]. Currently, 49 species of *Pinus* and 161 *Quercus* spp. are recognized in Mexico’s forests, placing the country as the second center of world diversity of both species [26, 27]. Temperate forests provide direct ecosystem services (wood, fiber, firewood) to about 18 million people who live in 9200 rural communities [24]. In addition, they offer more than 20 regulation and cultural ecosystem services that benefit many sectors of society [28, 29]. Biodiversity in temperate forests is imperative to sustain these ecosystem services. If biodiversity is jeopardized, its effects on the population level would be negative [30]. Hence its ecological and socio-economic importance.

In most temperate forests in northern Mexico, traditional forest management is based on the individual selection of trees [22]. Usually, the selection of trees to be removed is seldom based on all existing species. Only those of greater commercial value have a greater hierarchy and are preferred by forest managers [31]. Eventually, this type of harvesting changes the species composition and even the elimination of some of them in a particular site. Over time, many stands become unproductive for timber production and modify the order of provision of other ecosystem services. The purpose of this study was to evaluate the immediate effect of silvicultural practices on tree diversity that included intensive, semi-intensive, conservative, and no treatment (control group). The research questions were: what changes occur in tree diversity as a result of revised forest management schemes? How was the tree diversity status before and after the application of such practices? Which forest management practice does not affect tree diversity and still provides timber? We discuss the implications of the study’s results on the conservation of tree diversity in this type of ecosystem.

## Materials and methods

**Fig 1:**
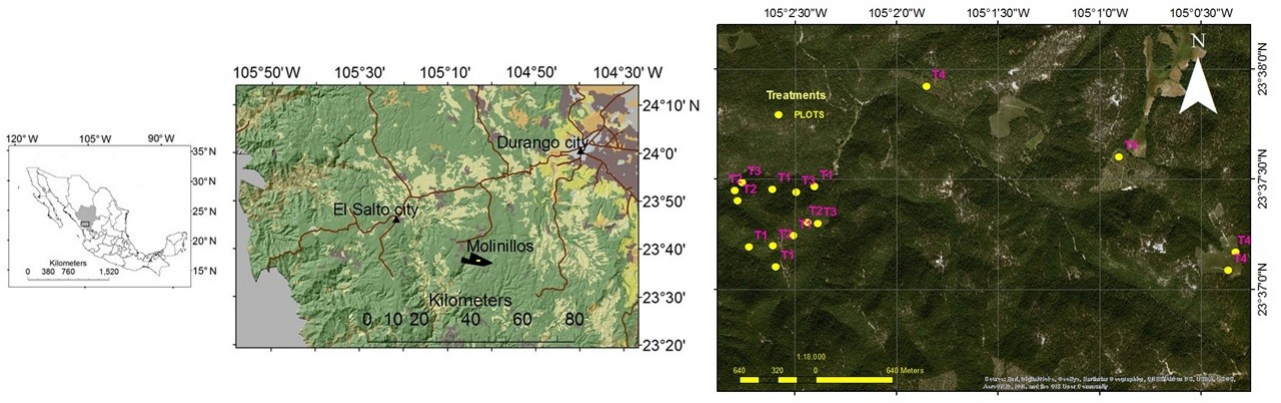
Location of the study area, Molinillos, in Durango, Mexico.

The study area is located in northern Mexico, within the Sierra Madre Occidental mountain range. Specifically, it is found in the property known as "Molinillos" in the southcentral part of the State of Durango (Fig 1). The Molinillos forestry ownership has a total area of 2866 ha, of which 2049 ha are under timber management. The owners allowed us to conduct the research in their property. These forests have been subject to timber management since the 1920s with the introduction of the Durango-El Salto railroad. In these early years, commercial interests prevailed where only the best and largest trees were harvested, with minimal investment in intermediate silvicultural treatments or protection and conservation activities [32]. Currently, the following treatments are applied in the area: clearcuts (12%), seed trees (5%), thinning (27%), and individual selection of trees (56%). A rotation period of 60-year and five 12-year cutting cycles were determined for the regular forest management plan [32]. The vegetation consists of temperate, oak-pine forests which are topographically distinguished by the presence of canyons, plateaus, and elevations of up to 2800 m above sea level [32]. The stands where the plots are located have similar climatic, ecological, and dasometric conditions.

The forest management scenarios evaluated were: an intensive treatment (T4) with removals of 100% of basal area (BA) or clearcut, a semi-intensive treatment (T3) with removals of 59–61% of BA and a conservative treatment (T2) with removals of 29–31% of BA. The last treatment was a control group with 0% removals (T1). The experimental plots were established in a simple random design with four repetitions for each treatment, except T4, which only had three repetitions, making it a total of 15. The letter G was used to identify the plots before treatment, while the letter T was used to identify them after said treatment.

The circular-shaped plots had a total area of 2,500 m^2^ (28.21 m radius) and a useful area of 1,000 m^2^ (17.84 m radius). The plots were established in October 2014, while the treatments were carried out in November 2015 and reassessed in October 2016. All tree species with a diameter at breast height (DBH)) ≥ 7.5 cm were identified and tallied. An enhanced forest management approach was applied to removals in T3 and T4 treatments. As opposed to the traditional approach, this one observed the following criteria: a) adhere to the targeted residual basal area, according to the treatment, b) leave a uniform spatial distribution of trees to ensure the protection of soil, c) eliminate sick, infested, or deformed trees, and d) maintaining, as much as possible, the diversity of species, so not one was preferred over the others. There was no limit in tree diameter for cutting.

### Information analysis

The plots were characterized using stand variables, before and after treatments, which included BA, canopy area, type of species, number of trees (abundance), and tree and total volume. For volume estimates, we used the functions generated for the forest region where the study area is located. These variables were used to determine any possible association with diversity indices. In the abundance sequences, the condition of the species from a greater to a lesser number was observed.

In order to assess the effects of management on tree diversity, in each plot we considered the following indicators: importance value index (IVI) by genus and species, alpha diversity, and similarity indices. The IVI is a structural index used to organize hierarchically the dominance of each species in mixed stands [33], and determines the vegetative hierarchy of each species or genus. The indices were calculated before and after applying the cuts specific to each forestry management treatment. The ecological importance value per tree species was determined using their abundance (number of individuals), dominance (as a function of the canopy area), and frequency (the number of individuals in each experimental plot) (Table 1).

**Table 1:** Equations for calculating Importance Value Index, alpha diversity indices, and evenness indices.

Indices	Description
Importance Value Index (IVI), IVI = RA+RD+RF	Relative abundance (RA) = (Absolute density of a species/Absolute density of all species) x 100
	Relative dominance (RD) = (Absolute dominance of a species/Absolute dominance of all species) x 100
	Relative frequency (RF) = (Absolute frequency of a species)/(Absolute frequency of all species) x 100
Alpha diversity	Species richness index (S) = total number of species
	Margalef's Index (D_mg_) = (S-1)/ln(N), where N = total number of individuals
	Simpson's diversity index (λ) = ∑pi, where pi = Proportional abundance of species (pi = ni/N) and ni = number of individuals of each species
	Shannon-Wiener index (H') = -∑pi ln pi
	Pielou's index (J') = H'/ln(*S*)
Evenness indices	Sørensen's Coefficient (S_I_) = 2*c*/(*a+b*), where *a* = number of species in plot *A*, *b* = number of species in plot *B*, *c* = number of species in plot *A* y *B*
	Jaccard Coefficient (J_I_) = *c*/(*a+b-c*)
	Quantitative Sørensen (S_Iquant_) = 2*pN/*(*aN + bN*), where *aN* = total number of individuals in plot *A*, *bN* = total number of individuals in plot *B*, *pN* = sum of the lowest abundance of each of the shared species A
	Morisita-Horn Index (M-H_I_) = (2∑(*an*_*i*_ *x bn*_*j*_))/((*da* + *db*) *aN* x *bN)*, *where an*_*i*_ = number of individuals of the i-th species in plot A, *bn*_*j*_ *=* number of individuals of the j-th species in plot B, *da* = ∑*an*_*i*_^*2*^ / *aN*^*2*^, *db* = ∑*bn*_*i*_^*2*^ / *bN*^*2*^

Source: [34–36].

The relative importance values are presented as percentages [34, 37–39]. Alpha diversity is a measure of species richness (i.e., number of species) that estimates the structure of the community (the proportional distribution of the value of importance of each species per plot) [35]. To quantify the specific richness, we used the species richness index (S) and Margalef's Index (Dmg). Community structure was estimated using Simpson's diversity index (λ), species evenness using Shannon-Wiener index (H'), and Pielou's index (J') [35, 36]. Also, we associated stand variables with alpha diversity using the Spearman coefficient, which is not readily influenced by atypical data [40].

Evenness indices express the degree of similarity among the same plots before and after treatment in terms of the species present. The indices that were estimated included qualitative (Sorensen's coefficient (SI) and Jaccard's coefficient (J_I_)), as well as quantitative (quantitative Sorensen's index (S_Iquant_) and Morisita-Horn index (M-H_I_)) [35]. The reason for using this set of indices was to ensure that all small differences between treatments were detected [36] (Table 1).

Before conducting the statistical analysis, we audited the data to check if they were normally distributed or met the statistical assumptions of linear regression models. The Shapiro-Wilkins and Levene's tests revealed that data followed a normal distribution and variances were equal for all variables and indices, except for the Margalef’s index. Therefore, the statistical analysis was done using both parametric and non-parametric methods. For instance, the Pearson and Spearman coefficients were used to determine the association between dasometric variables (e.g., basal area, number of trees, relative abundance) and diversity indices. The correlations and other statistical analysis were carried out using the SAS software package [40]. The significance level was established at *p* ≤ 0.05.

## Results

The tree strata in the sampling plots included 18 species, mainly pines, oaks, and madrones: *Pinus durangensis* Martínez, *P*. *arizonica* Engelm., *P*. *leiophylla* Schltdl. & Cham., *P*. *teocote* Schltdl. & Cham., *P*. *engelmannii* Carr., *P*. *lumholtzii* Rob. & Fern., *P*. *strobiformis* Engelm., *Quercus sideroxyla* Humb. & Bonpl., *Q*. *rugosa* Née, *Q*. *convallata* Trel., *Q*. *brachystachys* Benth., *Q*. *urbanii* Trel., *Q*. *durifolia* Seemen ex Loes., *Juniperus deppeana* Steud., *Arbutus madrensis* S. González, *A*. *tessellata* P.D. Sörensen, *A*. *arizonica* Sarg., and *Prunus serotina* Ehrh. These are grouped in five genera and five families: *Pinus* (Pinaceae), *Quercus* (Fagaceae), *Arbutus* (Ericaceae), *Juniperus* (Cupressaceae), and *Prunus* (Rosaceae). Three additional species were recorded at the border of the plots: *Pinus cooperi* Blanco, *Arbutus cf*. *arizonica*, and *A*. *bicolor* S. González, M. González, and P.D. Sörensen.

**Fig 2:**
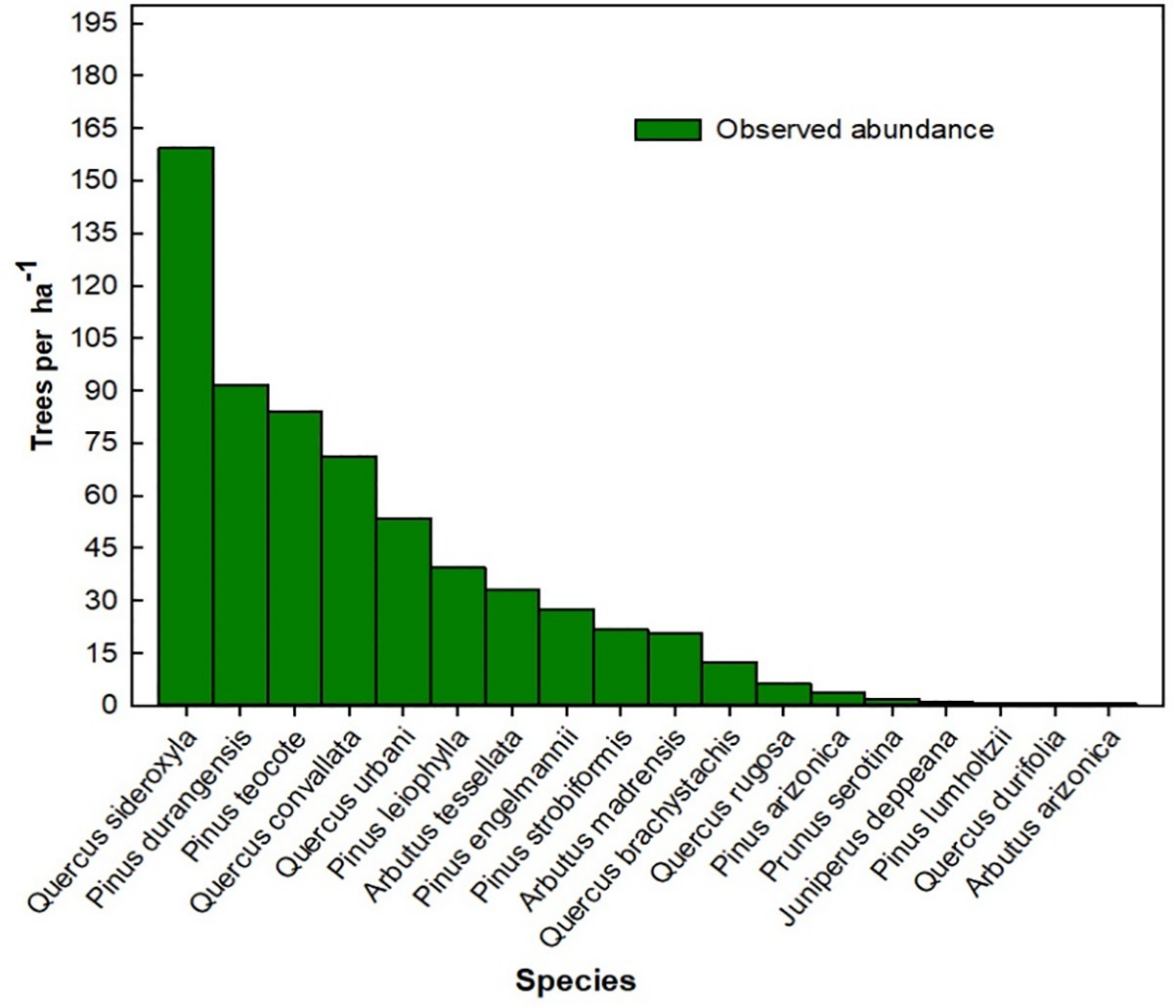
Abundance of tree species before applying silvicultural treatments in Molinillos, Durango, Mexico.

The average stand density and basal area in the sampling plots before the silvicultural treatments were 631 trees ha^-1^ and 18.96 m^2^ ha^-1^, respectively. The most abundant species were *Q*. *sideroxyla* (159 trees ha^-1^), *P*. *durangensis (*92 trees ha^-1^), *P*. *teocote* (84 trees ha^-1^), and *Q*. *convallata* (71 trees ha^-1^), whereas the scarcest were *Prunus serotina*, *Juniperus deppeana*, *P*. *lumholtzii*, *Q*. *durifolia*, and *A*. *arizonica*, with less than 4 trees ha^-1^ each (Fig 2).

The average DBH and total height, did not show statistically significant changes among treatments. The overall mean DBH was 17.3 cm before treatments and decreased to 16.1 cm after treatments. In contrast, basal area, canopy area, and total volume changed in accordance with the level of removal with each treatment (Table 2). The average total volume of removed trees (i.e., the difference between G-T) in the conservative treatment (T2) was 67.75 m^3^ ha^-1^, in the semi-intensive treatment (T3) 143.82 m^3^ ha^-1^, and in the intensive treatment (T4) 152 m^3^ ha^-1^.

**Table 2:** Stand variables before and after applying silvicultural treatments in a temperate forest in Durango, Mexico.

Stand variable	Before treatment (G)				After treatment (T)		
	G1: Control	G2: Conservative	G3: Semi-intensive	G4: Intensive*	T1: Control	T2: Conservative	T3: Semi-intensive
***Trees/ha***	805	645	665	747	805	500	368
***Average DBH (cm)***	16.40	17.78	17.67	14.89	16.40	16.61	15.29
***Maximum DBH (cm)***	64.70	55.80	79.10	56.20	64.70	53.00	40.70
***Minimum DBH (cm)***	7.50	7.50	7.50	7.50	7.5	7.5	7.5
***Average height (m)***	10.78	11.17	10.83	9.58	10.78	10.62	9.73
***Maximum height (m)***	30.00	29.90	28.00	30.00	30.00	29.90	27.30
***Minimum height (m)***	2.30	2.70	2.14	1.90	2.3	2.7	2.14
***Basal area (m***^***2***^ ***ha***^***-1***^***)***	23.20	21.18	22.07	14.12	23.20	14.49	8.65
***Canopy area (m***^***2***^ ***ha***^***-1***^***)***	12072	7490	9063	5706	12072	5653	3854
***Stem volume (m***^***3***^***)***	199.6	178.5	186.4	134.3	199.6	122.1	69.3
***Total volume (m***^***3***^ ***ha***^***-1***^***)***	231.3	210.9	223.1	152.0	231.3	143.1	79.3
***Cutting intensity (%)***					0	31.68	60.3

* In the intensive treatment (G4), all trees were removed. Thus, no data exist after treatments.

### Importance value index

**Fig 3:**
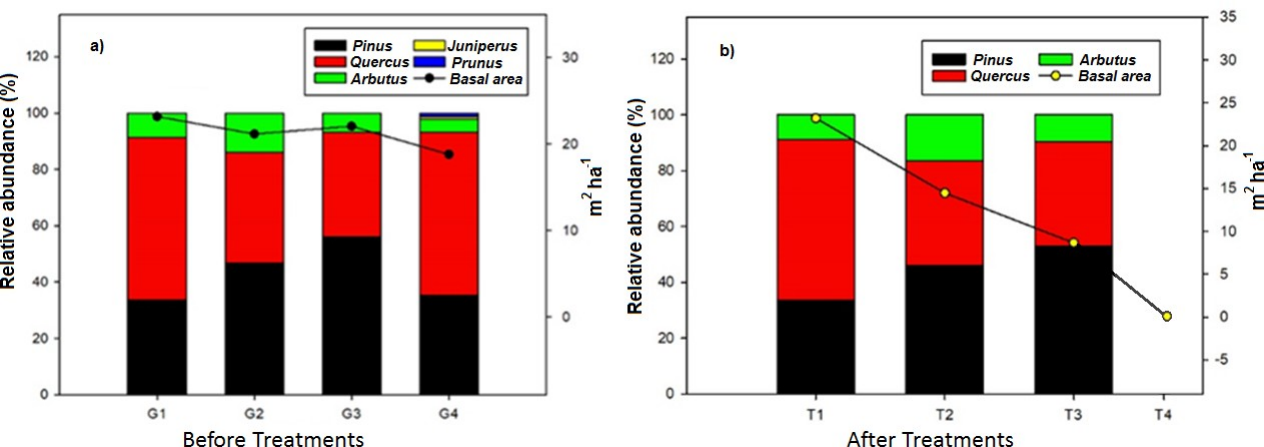
Relative abundance (%) and basal area per genus in a temperate forest in Durango, Mexico. The letters (a) and (b) indicate before and after treatments, respectively.

The absolute abundance (trees ha^-1^) and basal area (m^2^ ha^-1^) showed reductions that were statistically significant (abundance: *F*_(*p<*0.001)_ = 16.755; basal area: *F*_(*p<*0.001)_ = 13.346) (Fig 3). The species of *Pinus* and *Quercus* had the greatest abundance (Table 3). No significant changes due to treatment effects were recorded in the most abundant species (>50%): *Q*. *sideroxyla* (T1, T2, and T3), *Q*. *convallata* (T1), *P*. *durangensis* (T1, T2, and T3), *P*. *leiophylla* (*T3*), and *P*. *teocote* (*T2* and *T3*). The least abundant genus, before and after treatments, was *Arbutus* with less than 16%.

**Table 3:** Ecological values by genus, before and after treatment in a temperate-cold forest in Durango, Mexico.

Genus	Before treatment (G)								After treatment (T)							
		Abundance		Dominance		Frequency		IVI		Abundance		Dominance		Frequency		IVI
	BA	N	RA	CA	RD	AF	RF	%	BA	N	RA	CA	RD	AF	RF	%
**Treatment: Control, 0% removal**																
***Quercus***	10.70	465	57.8	5499	45.6	3.00	36.36	46.56	10.70	465	57.76	5499	45.55	3.0	36.36	46.56
***Pinus***	11.00	270	33.5	5918	49	4.00	48.48	43.68	11.00	270	33.54	5918	49.02	4.0	48.48	43.68
***Arbutus***	1.50	70	8.7	655	5.42	1.25	15.15	9.76	1.50	70	8.70	655	5.42	1.3	15.15	9.76
***Total***	23.20	805	100	12072	100	8.25	100	100	23.20	805	100	12072	100	8.25	100	100
**Treatment: Conservative, 29–31% removal**																
***Pinus***	12.51	303	46.9	4199	56.1	3.00	46.15	49.70	9.13	230	46	3693	58.62	2.5	41.67	48.76
***Quercus***	6.88	253	39.1	2586	34.5	2.25	34.62	36.09	4.42	188	37.5	1702	30.12	2.25	37.50	35.04
***Arbutus***	1.78	90	14	706	9.42	1.25	19.23	14.20	0.95	82.5	16.5	637	11.26	1.25	20.83	16.20
***Total***	21.18	645	100	7490	100	6.50	100	100	14.49	500	100	6032	100	6.00	100	100
**Treatment: Semi-intensive, 59–61% Removal**																
***Pinus***	13.86	373	56	5325	58.7	3.75	53.57	56.11	6.10	195	53.06	3193	64.62	3.25	52.00	56.56
***Quercus***	7.84	248	37.2	3463	38.2	2.00	28.57	34.67	2.28	138	37.41	1152	29.90	1.75	28.00	31.77
***Arbutus***	0.37	45	6.77	275	3.04	1.25	17.86	9.22	0.27	35	9.52	211	5.48	1.25	20.00	11.67
***Total***	22.07	665	100	9063	100	7.00	100	100	8.65	368	100	4557	100	6.25	100	100
**Treatment: Intensive, 100% Removal**																
***Pinus***	10.34	263	35.3	3029	53.1	3.33	40.00	42.79	0.0	-	-	-	-	-	-	-
***Quercus***	7.75	433	58	2188	38.3	2.67	32.00	42.78	0.0	-	-	-	-	-	-	-
***Arbutus***	0.61	33	4.46	321	5.63	1.33	16.00	8.70	0.0	-	-	-	-	-	-	-
***Juniperus***	0.06	7	0.89	41.9	0.73	0.67	8.00	3.21	0.0	-	-	-	-	-	-	-
***Prunus***	0.07	10	1.34	126	2.21	0.33	4.00	2.51	0.0	-	-	-	-	-	-	-
***Total***	18.82	747	100	5706	100	8.33	100	100	0.0	-	-	-	-	-	-	-

BA: Basal area (m^2^ ha^-1^), N: number of trees per ha, RA: Relative abundance (%), CA = canopy area (m^2^ ha^-1^), RD: Relative dominance (%), AF: Absolute frequency, RF: Relative frequency (%), IVI: Importance Value Index.

The dominant species (>35%) were *Q*. *sideroxyla* (T1, T3), *P*. *durangensis* (*T2*), *P*. *teocote* (*T2*, *T3*), and *P*. *strobiformis* (T1). Relative dominance of *Pinus* increased, while *Quercus* and *Arbutus* decreased after treatments. *Pinus strobiformis* (T2), *P*. *arizonica* (T3), and *Q*. *rugosa* (T3) were lost, as well as all species in T4. The least dominant species were *Arbutus arizonica* (T1), *P*. *leiophylla* (T2), and *Q*. *convallata* (T3). Canopy area (m^2^ ha^-1^) was statistically different between all treatments (*F*_(*p =* 0.002)_ = 9.940).

The relative frequency was relatively similar before and after treatments. The species with greater frequency were *Q*. *sideroxyla*, *Q*. *convallata*, *P*. *strobiformis*, *P*. *durangensis*, and *P*. *teocote*, while the least frequent were *Q*. *durifolia*, *P*. *lumholtzii*, *P*. *leiophylla*, *A*. *arizonica*, *J*. *deppeana*, and *P*. *serotina*.

The genera with the highest ecological importance values were *Pinus* (42–56%) and *Quercus* (34–46%), which did not generate significant changes after tree removal in treatments T2 and T3 (Table 3). Species with the greatest ecological value per plot before and after treatment were *Q*. *sideroxyla* (G3-T3) and *P*. *durangensis* (G2-T2), which had values between 18 and 24%. The least abundant species, that also had the least ecological value per plot before treatment (values less than 2.5%), were Q. *brachystachys* in G1, *P*. *leiophylla* in G2, *Q*. *convallata* in G3, and *Prunus serotina* in G4. After treatment, the least abundant species with values below 2% were *P*. *leiophylla* in T2 and *Q*. *convallata* in T3. Overall, the IVI values increased after treatment.

### Diversity and structure

**Fig 4:**
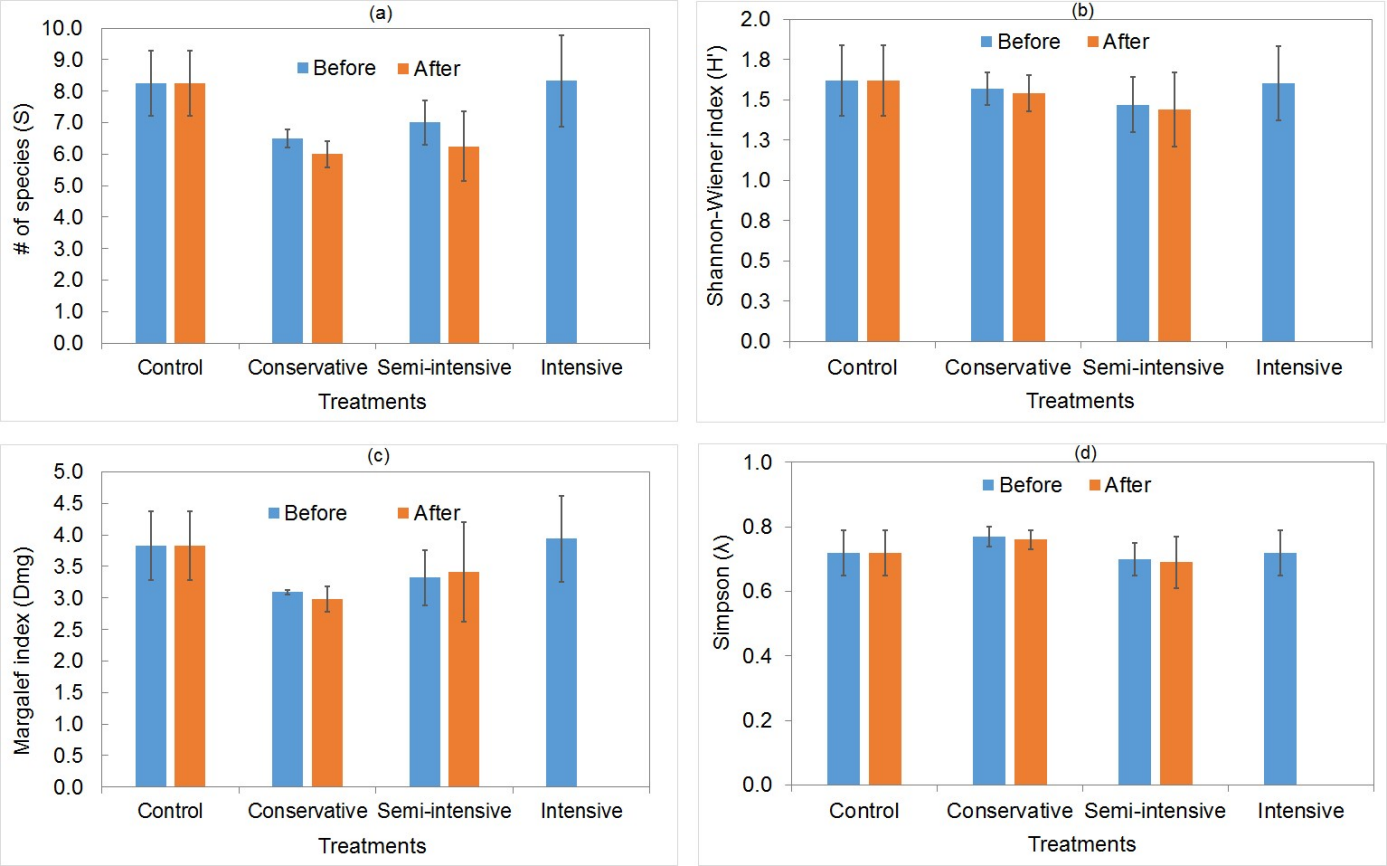
Alpha-diversity indices per treatment in a temperate forest in Durango, Mexico. The letters indicate: (a) Species richness, (b) Shannon-Wiener, (c) Margalef, and (d) Simpson indices.

Overall, the number of species before treatments decreased from 18 to 16, after treatments. Before applying treatments, the indices of diversity, dominance, species richness (S), Margalef (Dmg), Simpson (λ), Shannon-Wiener (H'), and Pielou (J') were not significantly different among plots (S: *F*_(*p =* 0.408)_ = 1.054; D_mg_: *χ*^*2*^_(*p =* 0.563)_ = 2.044; λ: *F*_(*p =* 0.897)_ = 0.196; H': *F*_(*p =* 0.934)_ = 0.141, and J': *F*_(*p =* 0.719)_ = 0.455). After the application of treatments T1, T2, and T3, they did not show significant changes either (S: *χ*^*2*^_(*p =* 0.223)_ = 2.999; D_mg_: *χ* ^*2*^_(*p =* 0.694)_ = 0.731; λ: *F*_(*p =* 0.804)_ = 0.223; H': *F*_(*p =* 0.821)_ = 0.202, and J': *F*_(*p =* 0.459)_ = 0.851). Clearly, the exception was T4 since there was a total elimination of tree diversity. Even though there were no significant differences among treatments (before and after application), it was possible to detect a small decrease in the values of species richness (S), Simpson (λ), and Shannon-Wiener (H') indices. Also, the Shannon-Wiener and the Simpson indices revealed that the conservative treatment had higher values than the semi-intensive treatment after cutting (Fig 4).

### Association between dasometric variables and diversity indices

Correlation coefficients revealed high associations among number of trees, canopy cover, and total volume. Diversity indices in general were not associated with stand variables, except the Pielou's evenness index, which was associated with number of trees, canopy cover, basal area, and total volume. The latter dasometric variable also exhibited some association with the Simpson’s diversity and Shannon-Wiener indices. In general, the association is negative, which suggests that tree diversity is lower in denser forests (Table 4).

**Table 4:** Correlation coefficients among stand variables and diversity indices in a temperate-cold forest in Durango, Mexico.

Stand variables	Basal Area	Canopy area	Total volume	Species richness (S)	Margalef Index (D_mg_)	Simpson's diversity (λ)	Shannon-Weiner (H')	Pielou's evenness (J')
**Num. of trees per ha**	0.751**	0.675**	0.644**	0.399	0.048	-0.101	-0.049	-0.427*
**Basal area (**m^2^ ha^-1^**)**	1	0.893**	0.970**	0.03	-0.249	-0.412	-0.358	-0.558**
**Canopy area (**m^2^ ha^-1^**)**	0.893**	1	0.884**	0.018	-0.273	-0.343	-0.314	-0.514*
**Total volume (**m^3^ ha^-1^**)**	0.970**	0.884**	1	-0.132	-0.396	-0.516*	-0.481*	-0.593**

** Significant at the 0.01 level

* significant at the 0.05 level. The alpha diversity indices were based on Spearman correlation. All others are based on Pearson correlation.

### Eveness indices

The evenness indices (Sorensen Coefficient (S_I_), Jaccard Coefficient (J_I_), Sorensen quantitative (S_Iquant_), and Morisita-Horn index (M-H_I_)) showed that the proportion of species decreased after the application of treatments (T2: 0.88 to 0.99 and T3: 0.71 to 0.96). In T1, the relationship remained the same (Table 5). According to Magurran (31), the Quantitative Sorensen is more robust to changes on the parameters, thus is the best index to explain species evenness. Based on this index, species composition decreased by 12% in the conservative management scenario, while in the semi-intensive scenario, it decreased by 29%.

**Table 5:** Evenness indices in a temperate-cold forest in Durango, Mexico.

Parameter	Evenness Indices			
	Qualitative		Quantitative	
	Sørensen's Coefficient (S_I_)	Jaccard Coefficient (J_I_)	Quantitative Sørensen (S_Iquant_)	Morisita-Horn Index (M-H_I_)
**Conservative management with removal of 29–31% BA (T2)**				
**Mean**	0.9575	0.9225	0.8825	0.99
**Minimum**	0.91	0.83	0.85	0.98
**Maximum**	1	1	0.94	1
**Standard error**	0.0246	0.0452	0.0202	0.00408
**Standard dev.**	0.04924	0.0903	0.0403	0.00816
**Semi-intensive management with removal of 59–61% BA (T3)**				
**Mean**	0.9275	0.875	0.7125	0.96
**Minimum**	0.8	0.67	0.66	0.93
**Maximum**	1	1	0.82	0.98
**Standard error**	0.0475	0.0792	0.0368	0.0108
**Standard dev.**	0.095	0.1584	0.0736	0.0216

## Discussion

The overall goal of this research was to evaluate the immediate effects of silvicultural treatments on species diversity in temperate forests. Our results suggest that in the short term, there were no significant changes in tree diversity in the conservative treatment (removal of 29–31% BA) and semi-intensive treatment (removal of 59–61% BA), notwithstanding the increase in total volume harvested. As mentioned earlier, tree removals were done based on new guidelines that included: a) observing the targeted residual basal area, b) leaving, as much as possible, a uniform spatial distribution of residual trees, c) eliminating sick, deformed, or dominated trees, and d) considering the diversity of species, so not one was favored over the others. The last two criteria contrast with traditional management plans, in which some pine species (e.g., *Pinus durangensis*, *P*. *cooperi*, *P*. *engelmanii*), are the most preferred due to their commercial value. These species usually receive priority in terms of reproduction, reforestation, and application of intermediate silvicultural practices that favor their growth. Our results imply that if silvicultural treatments are not directed towards a particular species, as it is usually done in the area, diversity is not modified substantially.

In general, our results do not agree with previous studies in similar ecosystems in which tree diversity did change because of forest management practices. For example, in a study conducted in the neighboring state of Chihuahua, authors found that tree diversity was greater in areas without treatment [38]. Although they reported that herbaceous and shrub species were more diverse in silvicultural treatments with seed trees and low intensity cuttings. Other studies have found that individual selection treatment maintain the number of tree species, although greater diversity was found in non-managed stands [41]. Likewise, Leyva-Lopez, et al. [42] found that the seed trees treatment, which resembles the semi-intensive treatment T3, did not have negative impacts on species composition and diversity, and even led to a slight increase on these indices. Differences in these results could be due to the type of successional stages, removal criteria, climatic conditions, and the evaluation time after applying the silvicultural treatments, among others.

No significant changes were observed in the species of *Pinus*, *Quercus*, and *Arbutus* when comparing relative abundance, relative dominance, and relative frequency. No significant changes were neither found before and after cutting in the conservative, semi-intensive, and no treatment management. Relative abundance ranged between 43% and 56% for *Pinus* and between 31% to 46% for *Quercus*, which contrast those values reported in studies with similar ecological forest conditions where the relative abundance of *Pinus* was above 68% and *Quercus* was below 35% [43, 44]. Apparently, low intensity cutting (such as the individual selection of trees or conservative treatments) implies little reduction in species abundance, low distribution variability, and a great homogeneity in the structure of oak-pine forests [45, 46].

Species with the highest importance values (IVI) in T1-T3 did not show significant changes before and after treatment. Selection cuts did result in the temporary loss of species which were those that had the least ecological value. These results are similar to those previously reported in areas with forest management where IVI ranged between 32% and 65% in *Pinus* species and less than 30% in *Quercus* species. They also coincided in other species with the least ecological value, such as *Juniperus sp*., *Prunus serotina*, and *Arbutus tesellata* (levels below 4%) [45, 47]. Ecological importance values are useful for monitoring the effect that cutting has and to prevent a decline in the species with the highest value and the potential loss of species with a low IVI value.

The specific richness (18 species) of the study area was similar or higher to that reported in studies of temperate forests in Mexico, particularly in Durango and Chihuahua [38, 47–49], Oaxaca [41, 42, 50], and Puebla [51]. Species richness indices did not show significant changes before and after cutting among plots. Species richness (S) varied from 6 to 10 species before and after treatment, while the Margalef (D_mg_) index varied from 2.9 to 3.8 (intermediate diversity). Both of which are higher than previously reported with Margalef reaching 0.79 to 1.58 in other forests of Durango and Chihuahua [38, 44, 46, 48, 49]. Regarding species diversity, Simpson's index (λ) showed intermediate to high diversity (0.69 to 0.77), which indicates that there is little possibility for a species to become dominant. Species evenness measured with the Shannon-Wiener's (H') index varied from 1.4 to 1.6 and Pielou's evenness (J') varied from 0.76 to 0.85. Other studies have reported similar results in ecologically equivalent areas with Shannon-Wiener varying between 0.34 and 1.58 and Simpson's Index between 1.36 and 2.97 [38, 44, 46, 48, 49].

There were no significant relationships among stand variables (basal area, canopy area, and number of trees) and alpha-diversity indices (Species richness (S), Margalef (D_mg_), Simpson (λ)). The exception was the Pielou's evenness index, which was negatively associated with number of trees, canopy cover, basal area, and total volume. This index suggests that tree diversity is lower in denser forests. Other authors have found that diversity indices increase as basal area increases [38, 49]. Perez-Verdin et al. [31] found a direct relationship between the Shannon-Wiener index and basal area up to 20 m^2^ ha^-1^, but after this density level, it started decreasing. This difference may be due to the careful selection of trees we conducted, considering the criteria exposed earlier.

Species evenness indicated levels greater than 88% in the conservative scenario and 71% in the semi-intensive one. The conservative treatment (i.e., removals of 29–31% of BA) did not modify the evenness substantially. Similar results have been reported by other authors when analyzing the effect of management in an area compared 30 years later where species evenness changed by 16% [38]. The results demonstrated that treatments T2 and T3 maintains species richness, although their abundance decreases.

Limiting factors for inferring results in other types of productive landscapes include time frames for measurement and the scale of the productive landscape assessed. We recommend expanding a permanent network of monitoring plots, allowing the inclusion of the widest ecological conditions possible (successional stages), dasometric characteristics, and varying forest management regimes so that the provision of ecosystem services are compatible. The indices to determine alpha diversity and evenness were selected to ease the calculation and interpretation of tree species abundance and composition.

### Implications for forest management

In order to preserve biodiversity, forest management must be diversified, and the traditional way in which trees are selected for removal that prevails today must be abandoned [31]. The management objectives must include maintaining the resistance (ability to stay unchanged despite the presence of disturbances) [52] and resilience (ability to absorb change and disturbance and still persist) [53]. Specific guidelines for assessing resistance and resilience have been proposed by Derose and Long [54]. The former is based on the influence of structure and composition of the ecosystem on disturbances. The latter, on the other hand, is measured through the influence of disturbances on the structure and composition of an ecosystem. Hence, structural and species diversity should be maintained or increased [12, 55].

According to Balvanera et al. [30] the effects of biodiversity change on processes are stronger at the community level, weaker at the ecosystem level, and negative at the population level. To address biodiversity effects at each level, and keeping both conservation of tree diversity and timber production as key objectives, we compiled a few recommendations based on literature and our own results: a) at the ecosystem level, stimulate the application of management regimes that increase landscape heterogeneity. Thom and Seidl reported that certain disturbances, caused by fire or bark beetles on ecosystem services, are generally negative, but they appear to favor biodiversity [55]. Thus, at this level, scattered, small patches of removed vegetation (through group-based selection of trees or small clearcuts) can be allowed. This patchy distribution can generate multi-specific stands with different ages that increase stand structural complexity [56]; b) at the community level, maintain or increase phenotypes and genotypes within the ecosystem [55]. The individual-based selection cut should consider all existing species and prioritize unhealthy, deformed, and dominated trees, preferably. In this study, the conservative treatment with 29–31% removal of basal area was compatible with the conservation of tree diversity (intermediate to high diversity, Shannon-Wiener Index = 1.54); and c) at the population level, planning and execution of the management regimes should be applied to all tree species, and not only to those that are of economic importance. Previously, it is necessary to determine proportional removals at the genus or species level to maintain species diversity and composition. Forest management plans should also consider the use of diversity indexes to identify less abundant species or those with low IVI values to prevent their elimination from harvesting sites. Likewise, those species in the opposite situation should be removed in proportion of their initial stocks.

To summarize, and to answer the research questions posed earlier, results imply that no significant changes in tree diversity are expected as a result of the application of either conservative or semi-intensive treatments. This also implies that both treatments are compatible with timber management and biodiversity conservation.

## Conclusions

This study was conducted to evaluate the immediate effect of silvicultural practices on tree diversity that included intensive, semi-intensive, conservative, and no treatment (control group) management. A total of 18 tree species, five genera, and five families were recorded in the study area. The species with the greatest ecological value were *Pinus durangensis*, *P*. *teocote*, *Quercus convallata*, and *Q*. *sideroxyla*. Results show that there were no significant changes before and after the application of conservative, semi-intensive, and without management scenarios on tree diversity in the study area when enhanced silvicultural practices are applied. The exception occurred in the clearcut treatment. The species evenness indices in the conservative forest regime maintained its species richness notwithstanding the changes in abundance. The relationship between stand variables and species diversity and richness was at best weak, suggesting that diversity is lower in denser forests.

In the short to mid-term, both the conservative and semi-intensive management scenarios allowed timber harvesting without generating significant changes on tree diversity. We recommend that the traditional way of selecting trees for removal should be changed, considering all existing species (commercial or not). We also recommend that those species with low IVI values be monitored to prevent them from being eliminated after applying silvicultural treatments. Proper identification and precise inventory of the species and stand variables should be implemented, being mindful of their ecological roles, and applying silvicultural treatments that avoid favoring only one group of species. Finally, a permanent monitoring network of forest resources in productive landscapes should be maintained [31, 57] and diversified. This would allow the monitoring of long-term impacts of silvicultural practices on landscape diversity and other ecosystem services, making appropriate adjustments for future forest management and planning.

## Supporting information

S1 Data (XLSX)

## References

[1] Armenteras D, González TM, Vergara LK, Luque FJ, Rodríguez N, Bonilla MA. Revisión Del Concepto de Ecosistema como “Unidad de La Naturaleza” 80 Años Después de su Formulación. Ecosistemas. 2016;25(1):83–9. 10.7818/ECOS.2016.25-1.12

[2] Montes C, Sala O. La Evaluación de los Ecosistemas del Milenio. Las relaciones entre el funcionamiento de los ecosistemas y el bienestar humano. Ecosistemas. 2008;16(3):137–47. 10.7818/re.2014.16-3.00

[3] MEA. Ecosystems and Human Well-being: Synthesis Millenium Ecosystem Assessment. World Resources Institute Island Press, Washington, DC 2005:137 p.

[4] Bennett EM, Peterson GD, Gordon LJ. Understanding relationships among multiple ecosystem services. Ecol Lett. 2009;12(12):1394–404. 10.1111/j.1461-0248.2009.01387.x 19845725

[5] Schuler L, Bugmann H, Snell R. From monocultures to mixed-species forests: is tree diversity key for providing ecosystem services at the landscape scale? Landscape Ecology. 2016 10.1007/s10980-016-0422-6

[6] Ammer C. Diversity and forest productivity in a changing climate. New Phytologist. 2019;221(1):50–66. 10.1111/nph.15263 29905960

[7] Isbell F, Calcagno V, Hector A, Connolly J, Harpole WS, Reich PB, et al High plant diversity is needed to maintain ecosystem services. Nature. 2011;477(7363):199–202. 10.1038/nature10282 21832994

[8] Quijas S, Schmid B, Balvanera P. Plant diversity enhances provision of ecosystem services: A new synthesis. Basic and Applied Ecology. 2010;11(7):582–93. 10.1016/j.baae.2010.06.009.

[9] Aguirre-Calderón OA. Manejo Forestal en el Siglo XXI. Madera y bosques. 2015;21:17–28.

[10] Barbier S, Gosselin F, Balandier P. Influence of tree species on understory vegetation diversity and mechanisms involved—A critical review for temperate and boreal forests. Forest Ecology and Management. 2008;254(1):1–15. 10.1016/j.foreco.2007.09.038.

[11] Brockerhoff EG, Barbaro L, Castagneyrol B, Forrester DI, Gardiner B, González-Olabarria JR, et al Forest biodiversity, ecosystem functioning and the provision of ecosystem services. Biodivers Conserv. 2017;26(13):3005–35. 10.1007/s10531-017-1453-2

[12] Triviño M, Pohjanmies T, Mazziotta A, Juutinen A, Podkopaev D, Le Tortorec E, et al Optimizing management to enhance multifunctionality in a boreal forest landscape. Journal of Applied Ecology. 2017;54(1):61–70. 10.1111/1365-2664.12790

[13] Dieler J, Uhl E, Biber P, Müller J, Rötzer T, Pretzsch H. Effect of forest stand management on species composition, structural diversity, and productivity in the temperate zone of Europe. European Journal of Forest Research. 2017;136(4):739–66. 10.1007/s10342-017-1056-1

[14] Sing L, Ray D, Watts K. Ecosystem services and forest management. Forestry Commission. 2015;Research note(9 2015):1–10.

[15] Duguid MC, Ashton MS. A meta-analysis of the effect of forest management for timber on understory plant species diversity in temperate forests. Forest Ecology and Management. 2013;303:81–90. 10.1016/j.foreco.2013.04.009.

[16] Zeller L, Liang J, Pretzsch H. Tree species richness enhances stand productivity while stand structure can have opposite effects, based on forest inventory data from Germany and the United States of America. Forest Ecosystems. 2018;5(1):4 10.1186/s40663-017-0127-6

[17] Chaudhary A, Burivalova Z, Koh LP, Hellweg S. Impact of Forest Management on Species Richness: Global Meta-Analysis and Economic Trade-Offs. Scientific Reports. 2016;6(1):23954 10.1038/srep23954 27040604 PMC4819217

[18] Paillet Y, Berges L, Hjalten J, Odor P, Avon C, Bernhardt-Romermann M, et al Biodiversity differences between managed and unmanaged forests: meta-analysis of species richness in Europe. Conservation biology: the journal of the Society for Conservation Biology. 2010;24(1):101–12. 10.1111/j.1523-1739.2009.01399.x 20121845

[19] Ranius T, Hämäläinen A, Egnell G, Olsson B, Eklöf K, Stendahl J, et al The effects of logging residue extraction for energy on ecosystem services and biodiversity: A synthesis. Journal of Environmental Management. 2018;209:409–25. 10.1016/j.jenvman.2017.12.048 29309965

[20] Wood A, Stedman-Edwards P. The Root Causes of Biodiversity Loss. 1st ed. London, UK: Routledge; 2000 10.4324/9781315071688

[21] Cordonnier T, Kunstler G, Courbaud B, Morin X. Managing tree species diversity and ecosystem functions through coexistence mechanisms. Annals of Forest Science. 2018;75 10.1007/s13595-018-0750-6

[22] Torres-Rojo JM, Moreno-Sánchez R, Mendoza-Briseño MA. Sustainable Forest Management in Mexico. Current Forestry Reports. 2016;2(2):93–105. 10.1007/s40725-016-0033-0

[23] Burke D, Elliott K, Holmes S, Bradley D. The effects of partial harvest on the understory vegetation of southern Ontario woodlands. Forest Ecology and Management. 2008;255:2204–12. 10.1016/j.foreco.2007.12.032

[24] Torres-Rojo J. Características de los núcleos agrarios forestales en México. In: Desarrollo Forestal Comunitario. 2015 p. 15–38.

[25] Rzedowski J. Diversidad y orígenes de la flora fanerogámica de México. Acta Botanica Mexicana. 1991(14):3–21.

[26] Gernandt DS, Pérez-de la Rosa JA. Biodiversidad de Pinophyta (coníferas) en México. Revista Mexicana de Biodiversidad. 2014;85:126–33. 10.7550/rmb.32195

[27] Valencia-Avalos S. Diversidad del Género Quercus (Fagaceae) en México. Boletín de la Sociedad Botánica de México. 2004;75:33–53.

[28] Perez-Verdin G, Sanjurjo-Rivera E, Galicia L, Hernandez-Diaz JC, Hernandez-Trejo V, Marquez-Linares MA. Economic valuation of ecosystem services in Mexico: Current status and trends. Ecosystem Services. 2016;21, Part A:6–19. 10.1016/j.ecoser.2016.07.003.

[29] Galicia L, Zarco-Arista AE. Multiple ecosystem services, possible trade-offs and synergies in a temperate forest ecosystem in Mexico: A review. International Journal of Biodiversity Science, Ecosystem Services and Management. 2014;10(4):275–88. 10.1080/21513732.2014.973907

[30] Balvanera P, Pfisterer AB, Buchmann N, He JS, Nakashizuka T, Raffaelli D, et al Quantifying the evidence for biodiversity effects on ecosystem functioning and services. Ecol Lett. 2006;9(10):1146–56. 10.1111/j.1461-0248.2006.00963.x 16972878

[31] Perez-Verdin G, Monarrez-Gonzalez JC, Tecle A, Pompa-Garcia M. Evaluating the multi-functionality of forest ecosystems in northern Mexico. Forests. 2018;9(4). 10.3390/f9040178

[32] Perez-Verdin G, Cassian-Santos JM, von Gadow K, Monarrez-Gonzalez JC. Molinillos private forest estate, Durango, Mexico In: Siry JP, Bettinger PS, Merry K, Grebner DL, Boston K, Cieszewski C, editors. Forest plans of North America. San Diego: Academic Press—Elsevier; 2015 p. 97–105. 10.1016/B978-0-12-799936-4.00013-8.

[33] Zarco-Espinosa V, Valdez-Hernández J, Ángeles-Pérez G, Castillo-Acosta O. Estructura y diversidad de la vegetación arbórea del Parque Estatal Agua Blanca, Macuspana, Tabasco. Universidad y ciencia. 2010;26:1–17.

[34] Mostacedo B, Fredericksen T. Manual de Métodos Básicos de Muestreo y Análisis En Ecología Vegetal. Santa Cruz, BOL: BOLFOR; 2000.

[35] Moreno EC. Métodos para medir la biodiversidad. Programa Iberoamericano de Ciencia y Tecnología para el Desarrollo. Oficina Regional de Ciencia y Tecnología para América Latina y el Caribe, UNESCO. 86 p. 2001. http://entomologia.rediris.es/sea/manytes/metodos.pdf. [Jan 24, 2020].

[36] Magurran AE. Measuring biological diversity Malden, MA: Blackwell Publishing Co; 2004.

[37] Alanís-Rodríguez E, Jiménez-Pérez J, Pando-Moreno M, Aguirre-Calderón ÓA, Treviño-Garza EJ, García-Galindo PC. Efecto de la restauración ecológica post-incendio en la diversidad arbórea del Parque Ecológico Chipinque, México. Madera y bosques. 2010;16:39–54.

[38] Hernández- Salas J, Aguirre-Calderón OA, Alanís-Rodriguez E, Jiménez-Perez J, Treviño-Garza EJ, González-Tagle MA, et al Efecto del manejo forestal en la diversidad y composición arbórea de un bosque templado del noroeste de México. Revista Chapingo Serie Ciencias Forestales y del Ambiente. 2013;19(2):189–99.

[39] Mora-Donjuán CA, Rubio-Camacho EA, Alanís-Rodríguez E, Jiménez-Pérez J, González-Tagle MA, Mata-Balderas JM, et al Composición y diversidad vegetal de un área de matorral desértico micrófilo con historial pecuario en el noreste de México. Polibotánica. 2014:53–66.

[40] Cook PA, Wheater P. Using Statistics to Understand the Environment. 1st ed. London, UK: Routledge, Taylor & Francis; 2000.

[41] Valdés M, Córdova J, Cárdenas M, Fierros A. Understory Vegetation and Ectomycorrhizal Sporocarp Diversity Response to Pine Regeneration Methods in Oaxaca, Mexico. Western Journal of Applied Forestry. 2003;18:101–8. 10.1093/wjaf/18.2.101

[42] Leyva-López JC, Velázquez-Martínez A, Ángeles-Pérez G. Patrones de diversidad de la regeneración natural en rodales mezclados de pinos. Revista Chapingo serie ciencias forestales y del ambiente. 2010;16:227–39.

[43] Jiménez-Bautista L, Damon A, Ochoa-Gaona S, Tapia RC. Impact of silvicultural methods on vascular epiphytes (ferns, bromeliads and orchids) in a temperate forest in Oaxaca, Mexico. Forest Ecology and Management. 2014;329:10–20. 10.1016/j.foreco.2014.05.053.

[44] Delgado Zamora DA, Heynes Silerio SA, Mares Quiñones MD, Piedra Leandro NL, Retana Rentería FI, Rodríguez Corral K, et al Diversidad y estructura arbórea de dos rodales en Pueblo Nuevo, Durango. Revista mexicana de ciencias forestales. 2016;7:94–107.

[45] Corral-Rivas JJ, Aguirre CO, Jiménez P, Corral RS. Un análisis del efecto del aprovechamiento forestal sobre la diversidad estructural en el bosque mesófilo de montaña "El Cielo", Tamaulipas, México. Investig Agrar Sist y Recur For. 2005;14(2):217–28. 10.5424/srf/2005142-00885

[46] Solís-Moreno R, Aguirre-Calderón OA, Treviño-Garza EJ, Jiménez-Pérez J, Jurado-Ybarra E, Corral-Rivas JJ. Efecto de dos tratamientos silvícolas en la estructura de ecosistemas forestales en Durango, México. Madera Bosques. 2006;12(2):49–64. http://www.redalyc.org/articulo.oa?id=61712205#.

[47] Graciano-Ávila G, Alanís-Rodríguez E, Aguirre-Calderón ÓA, González-Tagle MA, Treviño-Garza EJ, Mora-Olivo A. Caracterización estructural del arbolado en un ejido forestal del noroeste de México. Madera y bosques. 2017;23:137–46.

[48] Graciano-Ávila G, Aguirre-Calderón ÓA, Alanís-Rodríguez E, Lujan-Soto JE. Composición, estructura y diversidad de especies arbóreas en un bosque templado del Noroeste de México. Ecosistemas y recursos agropecuarios. 2017;4:535–42.

[49] Návar-Cháidez JdJ González-Elizondo S. Diversidad, estructura y productividad de bosques templados de Durango, México. Polibotánica. 2009:71–87.

[50] Luna-Bautista L, De La Rosa PH, Velázquez-Martínez A, Gómez-Guerrero A, Acosta-Mireles M. Understory in the composition and diversity of managed forest areas in Santa Catarina Ixtepeji, Oaxaca. Revista Chapingo, Serie Ciencias Forestales y del Ambiente. 2015;21(1):109–21. 10.5154/r.rchscfa.2014.08.037

[51] López-Hernández JA, Aguirre-Calderón ÓA, Alanís-Rodríguez E, Monarrez-Gonzalez JC, González-Tagle MA, Jiménez-Pérez J. Composición y diversidad de especies forestales en bosques templados de Puebla, México. Madera y bosques. 2017;23:39–51.

[52] Grimm V, Wissel C. Babel, or the ecological stability discussions: An inventory and analysis of terminology and a guide for avoiding confusion. Oecologia. 1997;109:323–334. 10.1007/s004420050090 28307528

[53] Holling CS. Resilience and stability of ecological systems. Annual Review of Ecology and Systematics. 1973;4:1–23.

[54] Derose RJ, Long JN. Resistance and resilience: a conceptual framework for silviculture. Forest Science. 2014;60(6):1205–12. 10.5849/forsci.13-507

[55] Thom D, Seidl R. Natural disturbance impacts on ecosystem services and biodiversity in temperate and boreal forests. Biological Reviews. 2016;91(3):760–81. 10.1111/brv.12193 26010526 PMC4898621

[56] Ehbrecht M, Schall P, Ammer C, Seidel D. Quantifying stand structural complexity and its relationship with forest management, tree species diversity and microclimate. Agricultural and Forest Meteorology. 2017;242:1–9. 10.1016/j.agrformet.2017.04.012.

[57] Zhao X, Corral-Rivas J, Zhang C, Temesgen H, Gadow Kv. Forest observational studies-an essential infrastructure for sustainable use of natural resources. Forest Ecosystems. 2014;1(1):8 10.1186/2197-5620-1-8

